# Postabortion Care: 20 Years of Strong Evidence on Emergency Treatment, Family Planning, and Other Programming Components

**DOI:** 10.9745/GHSP-D-16-00052

**Published:** 2016-09-28

**Authors:** Douglas Huber, Carolyn Curtis, Laili Irani, Sara Pappa, Lauren Arrington

**Affiliations:** a Innovative Development Expertise & Advisory Services, Inc. (IDEAS), Boxford, MA, USA; b United States Agency for International Development, Washington, DC, USA; c Population Reference Bureau, Health Policy Project, Washington, DC, USA; d Palladium, Health Policy Project, Washington, DC, USA; e University of Maryland, St. Joseph Medical Center, Towson, MD, USA

## Abstract

Worldwide 75 million women need postabortion care (PAC) services each year following safe or unsafe induced abortions and miscarriages. We reviewed more than 550 studies on PAC published between 1994 and 2013 in the peer-reviewed and gray literature, covering emergency treatment, postabortion family planning, organization of services, and related topics that impact practices and health outcomes, particularly in the Global South. In this article, we present findings from studies with strong evidence that have major implications for programs and practice. For example, vacuum aspiration reduced morbidity, costs, and time in comparison to sharp curettage. Misoprostol 400 mcg sublingually or 600 mcg orally achieved 89% to 99% complete evacuation rates within 2 weeks in multiple studies and was comparable in effectiveness, safety, and acceptability to manual vacuum aspiration. Misoprostol was safely introduced in several PAC programs through mid-level providers, extending services to secondary hospitals and primary health centers. In multiple studies, postabortion family planning uptake before discharge increased by 30–70 percentage points within 1–3 years of strengthening postabortion family planning services; in some cases, increases up to 60 percentage points in 4 months were achieved. Immediate postabortion contraceptive acceptance increased on average from 32% before the interventions to 69% post-intervention. Several studies found that women receiving immediate postabortion intrauterine devices and implants had fewer unintended pregnancies and repeat abortions than those who were offered delayed insertions. Postabortion family planning is endorsed by the professional organizations of obstetricians/gynecologists, midwives, and nurses as a standard of practice; major donors agree, and governments should be encouraged to provide universal access to postabortion family planning. Important program recommendations include offering all postabortion women family planning counseling and services before leaving the facility, especially because fertility returns rapidly (within 2 to 3 weeks); postabortion family planning services can be quickly replicated to multiple sites with high acceptance rates. Voluntary family planning uptake by method should always be monitored to document program and provider performance. In addition, vacuum aspiration and misoprostol should replace sharp curettage to treat incomplete abortion for women who meet eligibility criteria.

## Abstract (precis)

Twenty years of postabortion care (PAC) studies yield strong evidence that:

- Misoprostol and vacuum aspiration are comparable in safety and effectiveness for treating incomplete abortion.
- Misoprostol, which can be provided by trained nurses and midwives, shows substantial promise for extending PAC services to secondary hospitals and primary health posts.
- Postabortion family planning uptake generally increases rapidly-and unintended pregnancies and repeat abortions can decline as a result-when a range of free contraceptives, including long-acting methods, are offered at the point of treatment; male involvement in counseling-always with the woman’s concurrence-can increase family planning uptake and support.

## INTRODUCTION

Worldwide, 210 million women become pregnant annually; 135 million will have a live birth, and 75 million, or one-third, will have a spontaneous or induced abortion and need postabortion care (PAC). Of the 75 million abortions, 31 million are spontaneous (miscarriages) and 44 million are induced; half of the induced abortions are unsafe, performed by persons lacking the necessary skills or in an environment not in conformity with minimal medical standards.^1^

Since 1994, the United States Agency for International Development (USAID) has supported implementation of PAC programs in more than 40 countries to address complications related to miscarriage and incomplete abortion.^2^ PAC may be a unique service delivery model that is both curative and preventative—curative in treating incomplete abortion and the symptoms of hemorrhage and sepsis; preventative in providing family planning services to address unmet need for contraception and reduce unintended pregnancies and repeat abortions.

The 3 components of USAID’s PAC model are^2^:

1. Emergency treatment
2. Family planning counseling and service delivery, and where financial and human resources exist, evaluation and treatment of sexually transmitted infections (STIs) as well as HIV counseling and/or referral for testing
3. Community empowerment through community awareness and mobilization

USAID’s PAC model contains 3 components: emergency treatment, family planning, and community empowerment.

PAC is an integral part of maternity care, and all components of PAC services are essential, as stated at the 1994 International Conference on Population and Development. All 3 components have been included in USAID programs since 1994.^2^^,^^3^

In 2007, USAID published the first global PAC research compendium, “What Works, A Policy and Program Guide to the Evidence on Postabortion Care,” which reviewed the PAC literature from 1994 through 2003.^4^ This article summarizes the major findings on PAC interventions with strong evidence, described in the forthcoming second edition of USAID’s global PAC research compendium. The second edition builds on the first edition by including more detailed findings that address the cycle of repeated unintended pregnancy and abortion.^4^^-^^6^

## METHODS AND SCOPE

The first and second editions of the USAID PAC research compendium reviewed in detail more than 550 articles and reports published between 1994 and 2013, representing 20 years of PAC research.

We searched Scopus, MEDLINE, and POPLINE for relevant PAC interventions that had been evaluated, using several search words relevant to PAC, such as postabortion care, postabortion contraception, incomplete abortion, abortion complications, dilation and curettage, misoprostol, and manual vacuum aspiration. If the article title indicated there might be an intervention that could be replicated, we reviewed the full-text article to determine if there was sufficient information to be included in the “What Works” compendium and that the intervention took place in low- or middle-income countries in Asia, Africa, Latin America, or the post-Soviet states.^4^^,^^5^

Key interventions that had taken place only in high-income countries (namely Australia, Japan, Europe, or the United States) were included only if we determined that the interventions could be relevant for low-resource country contexts but had not yet been initiated in developing countries.

We also reviewed unpublished reports (gray literature) from key websites to supplement the studies published in peer-reviewed publications because of the limited amount of published literature on PAC. The websites reviewed comprised United Nations (UN) agencies, the Joint United Nations Programme on HIV/AIDS (UNAIDS), the World Health Organization (WHO), the Cochrane Collaboration, the Open Source Initiative (OSI), the International Center for Research on Women (ICRW), Futures Group, Population Services International (PSI), the Population Council, the International Council of Women (ICW), the World Bank, Family Health International (FHI) (renamed FHI 360 in 2011), Gynuity Health Projects, Ipas, and the Guttmacher Institute. Biomedical information was included insofar as it was relevant to programmatic considerations. The articles and reports included in our review covered the 3 components of the USAID PAC model as well as a fourth component that addressed policy, programs, and health systems in postabortion care. We adapted the Gray scale (Table 1) with its 5 levels to assess the strength of research evidence for each article or report cited in the compendium.^4^^,^^7^ Strong evidence was drawn primarily from the first 4 levels that included randomized controlled trials, well-designed trials with or without randomization, and some well-designed nonexperimental studies from more than one center or research group. Evidence was considered “strong” if it had support of at least 2 Gray I, II, or IIIa studies and/or 5 Gray IIIb, IV, or V studies.^7^

**TABLE 1:** Gray Scale of Strength of Evidence

Strength of Evidence	Description
I	Strong evidence from at least one systematic review of multiple well-designed, randomized controlled trials.
II	Strong evidence from at least one properly designed, randomized controlled trial of appropriate size.
IIIa	Evidence from well-designed trials/studies without randomization that include a control group (e.g., quasi-experimental, matched case-control studies, pre-post with control group).
IIIb	Evidence from well-designed trials/studies without randomization that do not include a control group (e.g., single group pre-post, cohort, time series/interrupted time series).
IV	Evidence from well-designed, nonexperimental studies from more than one center or research group.
V	Opinions of respected authorities, based on clinical evidence, descriptive studies, or reports of expert committees.

Note: Gray includes 5 levels of evidence. For the “What Works” compendiums, level III was subdivided to differentiate between studies and evaluations whose design included control groups (IIIa) and those that did not (IIIb).^101^ Qualitative studies can be classified as either level IV or V, depending on number of study participants and other factors. For more detail about these types of studies and their strengths and weaknesses, see Gray (2009).^7^

Studies with strong evidence for postabortion care are highlighted in this article and critical areas for future research are identified. Preference was given to studies from the Global South, but where evidence was scant from the South, studies from the Global North were included. Both editions of the compendium include a full description of the evidence statements along with complete references. In this article, we summarize the strong evidence we consider most applicable for extending PAC services, increasing access to family planning, improving cost-efficiency, and enhancing client satisfaction.

## FINDINGS

Recent PAC studies (2004–2013) reinforce most of the strong evidence statements from the 2007 PAC research compendium; specifically, those related to access to services, quality of care, community awareness of PAC, and postabortion family planning. Recent research also highlights the continuing challenges of providing complete PAC services, including family planning, counseling, and client information. Treatment options for incomplete abortion, task shifting, and decentralizing services are rapidly evolving and well documented.

In this section, we summarize key findings on the 4 main components covered in the scope of our literature review: (1) emergency treatment of incomplete abortion, (2) postabortion family planning counseling, including STI/HIV evaluation, (3) community empowerment, and (4) health systems and policies.

### Emergency Treatment of Incomplete Abortion

#### Surgical Treatment

Vacuum aspiration, either electric vacuum aspiration (EVA) or manual vacuum aspiration (MVA), is safer and less painful than sharp curettage, which is usually performed with general anesthesia in an operating theater.^4^ Many of the resources we reviewed focused on MVA. Main findings included:

- Women treated by vacuum aspiration (electric or manual) for incomplete abortion had shorter procedures and significantly less blood loss than those treated by sharp curettage. Pain is generally less than with sharp curettage.^4^
- The use of general anesthesia with sharp curettage is associated with increased risk of blood loss, cervical injury, uterine perforation, and subsequent abdominal hemorrhage.^4^
- Verbal support (“verbacaine”) to provide reassurance and diversion during the MVA procedure and/or paracervical block were generally inadequate for pain management with MVA.^8^^,^^9^ Sedation with analgesics plus emotional support during the procedure gave better results.^8^^,^^10^
- Paracervical block with local anesthesia for incomplete abortion with an open cervix provided no discernible benefit over placebo in studies of MVA procedures.^8^^,^^9^
- Costs can be substantially reduced by introducing MVA to replace sharp curettage when accompanied by changes in protocols for admission, place of procedure (often a simple procedure room), and an improved service delivery model that gives priority to PAC clients. Direct costs are reduced by avoiding use of the operating theater, general anesthesia, blood transfusions, and prolonged hospital stays.^11^^-^^13^
- Prophylactic antibiotics to prevent postoperative infection have not shown effectiveness in the relatively underpowered studies from the Global South.^14^^,^^15^ One large study of induced abortion in the United States showed a reduction in postoperative infections for vacuum aspiration and medical induced abortion using doxycycline 100 mg twice daily for 7 days.^16^ However, infections were rare with or without prophylactic antibiotics.

Electric and manual vacuum aspiration are safe, effective, and preferable to sharp curettage for uterine evacuation.

#### Medical Management

The past decade has seen major advances in medical evacuation using misoprostol for treatment of incomplete abortion. Misoprostol is rapidly gaining acceptance as a safe, effective treatment for incomplete abortion in primary and secondary outpatient settings and can be provided by trained nurses and midwives. It should be an available option for women needing postabortion care.

Misoprostol is safe and effective for treatment of incomplete abortion and can be provided by trained nurses and midwives.

Evidence from at least 16 studies of misoprostol points to an optimal dose and route of administration of 400 mcg sublingually or 600 mcg orally; both are equally effective for treating uncomplicated first-trimester incomplete abortion, either spontaneous or induced. No advantage was seen with higher or multiple doses, or when given by other routes of administration.^5^

Study criteria for misoprostol treatment typically included first-trimester incomplete abortion without complications and an open cervix. Other selection criteria in clinical trials usually included women residing within one hour’s travel distance of a hospital. Back-up surgical evacuation and ultrasound were available on site or by ready referral to another regional or tertiary hospital.

Table 2 summarizes findings from 8 high-quality studies conducted in the Global South between 2005 and 2012 in tertiary and district hospitals.^14^^,^^17^^-^^23^ Successful evacuation rates ranged between 89% and 99% at 1 to 2 weeks post-treatment without recourse to surgical intervention. A follow-up was usually scheduled at 1 week. If evacuation was not complete, the woman was offered the option of waiting a second week or having an immediate surgical evacuation, usually by MVA. If not complete at 2 weeks, a surgical evacuation was performed.

**TABLE 2:** Effectiveness and Satisfaction With Treatment for Incomplete Abortion, Misoprostol Compared With Surgical Evacuation, 10 Countries, 2005–2012

Article	Country (Sample Size)	Study Design: Misoprostol/ Surgical Comparison Group	Effectiveness: % With Complete Evacuation	% Client Satisfaction With Procedure	Comments
Blandine 2012^17^	Burkina Faso (N = 99)	400 mcg misoprostol sublingually/ referral for surgical	M: 98%	M: 99%	PAC with misoprostol introduced to 2 district hospitals with no previous PAC service. All eligible women chose misoprostol over optional referral for MVA.
Dao 2007^18^	Burkina Faso (N = 447)	600 mcg misoprostol orally/MVA	M: 94% S: 99%	M: 97% S: 98%	2 teaching hospitals.
Weeks 2005^14^	Uganda (N = 317)	600 mcg misoprostol orally/MVA	M: 96% S: 92%	M: 94% S: 95%	Misoprostol was associated with less pain and fewer complications but increased bleeding. All received antibiotics after treatment.
Taylor 2011^19^	Ghana (N = 230)	600 mcg misoprostol orally/MVA	M: 98% S: 99%	M: 94% S: 89%	44% were very satisfied with misoprostol vs. 8% with MVA; 95% of those treated with misoprostol would choose it again vs. 36% treated with MVA.
Shwekerela 2007^20^	Tanzania (N = 150)	600 mcg misoprostol orally/MVA	M: 99% S: 100%	M: 99% S: 100%	75% were very satisfied with misoprostol vs.55% with MVA; more side effects were associated with misoprostol; greater pain with MVA.
Bique 2007^21^	Mozambique (N = 270)	600 mcg misoprostol orally/MVA	M: 91% S: 100%	M: 96% S: 100%	87% were very satisfied with misoprostol vs. 37% with MVA; trained midwife provided MVA with only verbal anesthesia; tertiary hospital site.
Montesinos 2011^22^	Ecuador (N = 242)	600 mcg misoprostol orally/MVA	M: 94% S: 100%	M: 96% S: 97%	47% were very satisfied with misoprostol vs. 40% with MVA; ultrasound use decreased threefold for misoprostol and MVA in 1 year.
Shochet 2012^23^	Senegal (N = 199) Niger (N = 152) Mauritania (N = 119) Nigeria (N = 51) Burkina Faso (N = 318)	400 mcg misoprostol sublingually/ standard surgical care (MVA or D&C)	*Senegal* M: 93% S: 100% *Niger* M: 89% S: 100% *Mauritania* M: 91% S: 100% *Nigeria* M: 96% S: 100% *Burkina Faso* M: 98% S: 100%	*Overall in the 5 countries* M: 99% S:98%	Antibiotics given with the surgical option; success rates much higher with misoprostol after first month from introduction. Ultrasound not needed on site. Nurses and midwives had prominent roles in care in Burkina Faso, Niger, and Senegal.

Abbreviations: D&C, dilation and curettage; M, misoprostol; MVA, manual vacuum aspiration; PAC, postabortion care; S, surgical.

Successful evacuation rates with misoprostol ranged 89%–99% at 1–2 weeks post-treatment.

Success rates were lower in studies with shorter time frames for judging completion and more frequent follow-up visits. For example, one study scheduled the first follow-up visit at day 2; if evacuation was not complete, women could opt to return at day 7, but not later. Success rates were lower and also different between the 2 study sites, 44% vs. 85%, suggesting major provider and institutional variations. The authors of the study acknowledged that providers’ lack of confidence in using misoprostol may partly explain the large differences between sites.^24^

Surgical evacuation is still needed for the 2% to 8% of women with incomplete evacuation following misoprostol; women presenting with complications (e.g., sepsis, heavy bleeding); women with advanced gestational age; and women who prefer surgical management.^25^^-^^27^ Most studies specified that participants live within the facility’s geographic catchment area or within one hour of travel time, and that ultrasound be available by referral. Ultrasound was seldom needed; current misoprostol training curricula note that ultrasound is not required to diagnose complete evacuation, although in selected circumstances it may assist in the evaluation.^27^ None of the 8 misoprostol studies in Table 2 documented postabortion contraception uptake, a critical component of postabortion care.

Recent programmatic expansions in Senegal document favorable client and clinical outcomes when misoprostol is given at lower-level health facilities farther from hospitals.^25^ Thirteen studies in 9 countries included nurse-midwives as providers.^14^^,^^17^^,^^18^^,^^21^^,^^23^^,^^25^^,^^28^^-^^34^

Major findings with misoprostol for PAC are as follows:

- Success rates were comparable with MVA in most studies using doses of 400 mcg sublingually or 600 mcg orally with optional follow-up extended to 2 weeks (Table 2). Surgical evacuation (usually MVA) was available on site or by referral if the woman requested and for those who did not meet the criteria for misoprostol.
- Complications were low and comparable to MVA. Most studies reported less pain with misoprostol than with MVA.^14^^,^^20^ Bleeding was slightly increased, compared with MVA, though not of clinical significance.^14^ Client acceptability was high, with substantially more women reporting they were “very satisfied” compared with MVA. Misoprostol recipients were also more likely to recommend it to a friend (Table 2).
- High client satisfaction and complete evacuation rates were also achieved when misoprostol was expanded to secondary and district hospitals and health posts that had no previous capability to treat incomplete abortion.^17^^,^^23^^,^^25^^,^^28^^,^^29^
- Pain management with ibuprofen, 800 mg to 1000 mg, was generally adequate and clearly superior to paracetamol (acetaminophen).^35^^-^^37^
- Routine ultrasound was not necessary to diagnose incomplete abortion or confirm complete uterine evacuation when misoprostol was used at less than 12 weeks gestation; clinical judgment was adequate for the large majority of cases.^14^^,^^17^^,^^20^
- Two studies noted that ultrasound available through referral was helpful for establishing ectopic pregnancy, determining gestational age, and confirming complete evacuation for a few cases.^17^ Typically ≥90% of women in Burkina Faso and Senegal were managed entirely at the primary point of care without referral for ultrasound.^14^^,^^17^^,^^20^ However, these studies contained few sites.
- Mid-level clinicians can correctly diagnose and treat incomplete abortion with misoprostol and accurately determine complete evacuation.^23^^,^^25^^,^^28^ Expanding the scope of practice for mid-level providers for counseling and provision of misoprostol was found to be safe, effective, and acceptable.^17^^,^^18^^,^^21^

### Postabortion Family Planning Counseling and Services

Although emergency treatment for incomplete abortion often receives greater attention than postabortion family planning, the family planning component to reduce preventable child and maternal deaths in both the postpartum and postabortion periods is a growing focus for maternal, neonatal, and child health programs.^1^^,^^38^ Many PAC clients were not using a modern contraceptive method at the time of conception. When providing family planning counseling and services, it is necessary to understand the reproductive intentions of the woman and her partner. Expanding the method mix, including long-acting reversible contraceptives (LARCs) and permanent methods, allows the woman to choose what she wants and what works best for her. Women need adequate counseling and choice of methods to make informed voluntary decisions about family planning.

The Family Planning High Impact Practices brief on postabortion care summarized the results of 15 studies to strengthen postabortion family planning in 14 countries.^39^ Postabortion family planning uptake before discharge rose by 30–70 percentage points within 1 to 3 years in several studies, sometimes increasing by as much as 60 percentage points in 4 months.^4^^,^^39^ The interventions providing postabortion family planning counseling and services at the same time and location as treatment rapidly increased immediate postabortion contraceptive acceptance. They found an average 37 percentage point increase of women receiving a method before discharge, from 32% before the intervention (range, 1% to 65%) to 69% post-intervention (range, 25% to 98%).^39^ Other post-intervention studies reported even greater family planning uptake.^40^ Counseling and family planning uptake continued to increase in one Peru study over 3 years after support for the intervention stopped, a demonstration of sustainability.^41^ One study in Zimbabwe comparing a postabortion family planning intervention at one hospital with a control hospital with no special intervention reported significantly greater family planning uptake in the intervention hospital, as well as significantly fewer unintended pregnancies and repeat abortions; the study enrolled 982 women in total, 527 of whom were followed for 12 months.^42^

Postabortion family planning uptake before discharge increased by 30–70 percentage points in 1–3 years when family planning services included a wide range of methods provided at the same time and location as treatment.

Multiple studies (published between 1998 and 2010) from 9 countries show that training providers in family planning counseling, service delivery, and MVA increases family planning uptake.^39^^,^^41^^,^^43^^-^^46^ Other factors contributing to success were continuing education in family planning counseling skills, provider job aids, and client education materials.^47^^,^^48^ Placing commodities at the point of treatment such as in a wall cabinet in the treatment room also increased performance, as did supportive supervision.^47^^,^^49^

After women gave permission for including their husbands in counseling, family planning use increased. Counseling covered follow-up care, rapid return to fertility, and family planning methods. Men also provided physical, emotional, and material support for PAC clients during recovery. Husbands who were counseled were 60% more likely to provide a high level of support for family planning use; 30% were also more likely to provide a high level of emotional support.^50^^-^^53^

Providing all contraceptive methods free to PAC clients greatly contributed to the increased uptake of postabortion family planning prior to discharge from the facility.^42^^,^^46^^,^^48^^,^^49^^,^^54^ This is a key consideration for governments, ministries of health, and policy makers. Many women are not able to pay for contraceptives at the time of treatment.

Couples counseling, with approval from the woman, improved family planning uptake and support.

Providing all contraceptive methods free greatly increased uptake of postabortion family planning because many women cannot pay at the time of treatment.

Delaying the next pregnancy for at least 6 months after miscarriage or abortion reduces the incidence of preterm delivery, low birth weight, premature rupture of membranes, and maternal anemia.^55^ Therefore, it is particularly important to stress the need for spacing after spontaneous abortion when women may desire another pregnancy soon. Advising every woman to delay her next pregnancy also removes the potential stigma that accepting family planning implies her abortion was induced.

Insertion of an intrauterine device (IUD) immediately after a first-trimester abortion yielded few expulsions and carried no increased risk of perforation or infection. There was only a minimal increase in expulsion rates over delayed insertion.^56^^,^^57^ With misoprostol treatment, the IUD is usually inserted at the 1-week follow-up visit provided that uterine evacuation is complete.^57^ Delayed insertion may increase the likelihood of failing to return for the planned IUD insertion, thus placing women at higher risk of unintended pregnancy.^58^

Several studies found that women receiving immediate postabortion LARCs had fewer subsequent unintended pregnancies and repeat abortions than those who were offered delayed insertions (3 to 6 weeks post-procedure).^56^^,^^59^^,^^60^

#### HIV Testing and STI Prevention

Women with HIV who are symptomatic are at an increased risk for spontaneous abortion.^61^^-^^65^ Few studies have been done in this area of PAC and HIV, although some show promising results. For example, in Tanzania offering HIV testing to 706 women as part of PAC services resulted in 58% of the women accepting HIV testing; 14% of these women had HIV. HIV testing in conjunction with PAC was found to be acceptable and relevant in this high-risk setting.^66^

We did not find documentation that PAC services routinely included counseling on STI and HIV risk factors and provided condoms for prevention. One study identified this as an unmet need.^67^ No studies were found that documented both diagnosis and treatment for STIs and HIV in relation to PAC.

#### Other Counseling Concerns

Women who have undergone induced or spontaneous abortion may require special counseling considerations. Increased risk of spontaneous abortion is associated with several common conditions, including malaria, HIV/AIDS, STIs, gender-based violence, smoking, excess alcohol use, exposure to pesticides, and previous spontaneous abortion.^68^^-^^75^ Approximately one-third of women seeking abortion have been victims of abuse sometime during their lifetime.^73^^,^^76^ Significant anxiety is experienced by 40% to 45% of women before an induced abortion. Distress is reduced after the procedure, although about one-fourth may report anxiety and depression from 1 month to 2 years after the event. The long-term psychological outcomes are similar between women who have had an induced abortion and those who have given birth.^5^^,^^77^^,^^78^ The short-term emotional reactions to miscarriage appear to be as large or larger than those to induced abortion.^5^^,^^6^ Most psychological outcome studies, however, have been conducted in developed countries, so it is difficult to apply the findings to low-resource settings. Some of women’s counseling needs may be amenable to community education and intervention as part of community health worker (CHW) activities. However, studies are not yet available to document interventions and effectiveness.

About one-third of women seeking abortion have been victims of abuse sometime during their lifetime.

A substantial percentage (40%) of PAC clients are under the age of 25, yet they account for almost half of the deaths from unsafe abortion.^79^ Factors contributing to maternal deaths in young PAC clients include not seeking PAC services soon enough due to late recognition of complications, delay due to stigma in the community, lack of funds for transport and services, and delay due to provider bias and attitude to young clients.^80^^,^^81^ Skilled counseling is especially important for younger women whose first interaction with the health system may be for postabortion care.^1^

### Community Awareness, Mobilization, and Empowerment

The Community Action Cycle—a participatory problem-solving approach involving community diagnosis, planning, implementation, participatory evaluation, and scale-up—can increase awareness of PAC services, especially through training of CHWs to provide postabortion family planning counseling and services, including referral to facilities.^82^ In Kenya, CHWs were instrumental in rapidly increasing access to essential PAC services by providing contraceptives to 63% of users and referring 90% of PAC clients to health facilities.^83^^,^^84^ The Community Action Cycle also helped educate communities on recognition of danger signs during pregnancy, such as bleeding, to encourage health-seeking behavior for PAC.^85^ However, major gaps remain in knowledge of linkages between community mobilization and PAC services.

### Policy, Programs, and Systems

#### Wide Replication of PAC Including Essential Organization of Services

In several countrywide PAC programs, postabortion family planning is emerging as a strong program component.

Nepal was one of the first countries to widely replicate PAC programming and document valuable lessons learned during the expansion phase. The lessons are especially relevant as the challenges are similar throughout most PAC programs. In 1995, Nepal first initiated PAC services at the Maternity Hospital in Kathmandu. It then expanded services to 78 sites in 50 districts mainly in public-sector health facilities.^85^ The program focused on (1) replacing sharp curettage with MVA by establishing PAC training centers at the district level using group-based and on-the-job training, and (2) training staff nurses and senior auxiliary nurse-midwives as primary providers, thereby expanding access to primary health centers and linking PAC with family planning services. Key achievements included training more than 170 providers in PAC and starting 46 new PAC service sites in primary health centers with task shifting for most of PAC services from doctors to nurses. This resulted in the increase of MVA use from 47% to 74% and family planning uptake of 81% (a high percentage compared with most programs although baseline data were not available), with injectables being the most popular method.^86^

In Nepal, technical support service centers were crucial for institutionalizing comprehensive PAC services. Resistance by doctors and hospital management to nurses providing MVA was partly overcome through formal and informal orientations for doctors. Structured on-the-job training was cost-effective for maintaining PAC skills and strengthening services. PAC program data were difficult to collect as they were not included in the Ministry of Health and Population’s regular health management information system. Challenges included the frequent transfer of trained PAC service providers; unavailability of family planning services to women having sharp curettage procedures; insufficient caseload at some sites to support group-based training; and irregular supply and replacement of MVA equipment.^86^

Guatemala expanded its PAC program to 22 of the 33 public district hospitals. The 22 district hospitals increased postabortion family planning counseling from 31% to 78% over 18 months; postabortion family planning acceptance before discharge rose from 20% to 49%; and vacuum aspiration increased from 38% to 68%.^49^

Tanzania achieved high performance when initiating PAC with MVA, expanding from 11 to 229 sites between 2005 and 2010. In 2010, the PAC program served 9,563 PAC clients, with 84% of women being counseled on family planning and 82% accepting a method.^2^ Senegal also rapidly expanded services in 23 of 56 districts, covering 60% of the population by 2006. Formal PAC training was given to 523 providers from 323 health facilities. Health centers with adequate space, materials, and equipment increased from 29% to 95% between 2003 and 2006, and facilities with reorganized services capable of providing 24/7 PAC services rose from 55% to 84%.^87^

In replicating PAC services, Rwanda engaged lower-level health facilities to provide PAC, including misoprostol, and increased use of family planning services. Before the pilot program, only 2 of 50 health centers were providing PAC services; all other health centers referred PAC clients to hospitals. Within a year, 91% of PAC clients were treated at health centers and only 9% were referred to hospitals. Overall, misoprostol was used to treat 83% of PAC clients, and 59% of women were discharged with a contraceptive method.^29^ A system of data collection and monitoring was implemented to inform the scale-up, providing a good example of the inclusion of family planning uptake in the health management information system.^29^ Wide replication of PAC in multiple sites is a strong step toward sustainable and scaled-up services. These are not as well documented in the literature.

#### Task Shifting

An expanded scope of practice for mid-level providers was assessed in a number of studies providing PAC service delivery. These studies confirm the safety, effectiveness, and acceptability of trained nurses and midwives as providers of MVA, affirming their ability to accurately determine complete evacuation.^23^^,^^28^^,^^88^^,^^89^

#### Costs

Twelve studies compared costs for PAC services using MVA versus sharp curettage. There was strong evidence that using MVA for PAC instead of sharp curettage along with changes in protocols and an improved service delivery model can significantly reduce costs of care in most cases.^4^^,^^5^ In-patient hospital and personnel costs were the main drives of cost for PAC service delivery. Interventions to reduce costs included^4^^,^^5^:

- Changes in protocol to the standard use of MVA with local anesthesia if the uterus was smaller than 12 weeks’ size
- Providing PAC as an ambulatory outpatient procedure
- Provider training on information and counseling (on health status, uterine evacuation procedure, postabortion contraception, and care after leaving the hospital)
- Refresher training and supportive supervision

Using MVA instead of sharp curettage can significantly reduce costs of care in most cases.

A number of cost studies document the high cost of treating abortion complications, the value of expanding PAC services with a robust family planning component, and the substantial savings that could be achieved by preventing unintended pregnancies and abortion through improved contraceptive services.^90^^-^^95^ Immediate postabortion insertion of an IUD after surgical evacuation offers special promise of cost-efficient, high contraceptive continuation with a very effective method.^96^

One study also made the case that greater use of misoprostol for PAC could free surgical resources to be used for more complicated cases.^34^ However, good costing studies on use of misoprostol compared with surgical evacuation are lacking; this is a major area for further research and documentation.

## PAC PROGRAM AND PRACTICE RECOMMENDATIONS

The implications for practice flow from PAC research documenting practices that impact health outcomes and that are practical and cost-efficient to implement. These include emergency treatment of incomplete abortion and postabortion family planning.

### Emergency Treatment of Incomplete Abortion

Medical (misoprostol) and surgical management (electric or manual vacuum aspiration) have been proven to offer safer options over sharp curettage for treatment of incomplete abortion. In countries where both misoprostol and vacuum aspiration are authorized, women should be informed of appropriate treatment options. Programs should ensure that both are available, staff are trained, and communities are informed, including CHWs. New protocols and training curricula for vacuum aspiration and medical evacuation, for example, from USAID and Ipas, incorporate the findings of recent research and will facilitate revised practices.^26^^,^^27^^,^^97^^,^^98^ These are important instruments for ensuring quality and competence of providers and programs.

### Postabortion Family Planning

The voluntary informed choice of whether to use contraception must always be solely that of the woman. Asking the woman about her reproductive intentions and providing counseling for all methods (short-acting methods, LARCs, and permanent methods) will promote informed and voluntary choice for her; her health and that of her next pregnancy will benefit. Several studies have found an urgent need to improve content and quality of client counseling. The most important recommendations emerging from PAC studies are to offer all women family planning counseling and services before they leave the facility regardless of the method of uterine evacuation, and to complement counseling with written instructions on:

1. Her chosen contraceptive method—including how to use the method, what to expect, and how to resupply
2. The rapid return of fertility after the abortion (within 2 to 3 weeks)
3. The signs and symptoms of postabortion complications (e.g., heavy bleeding, fever, increasing pain) that would need medical attention

All postabortion women should receive family planning counseling and services before they leave the facility.

These actions are endorsed by a joint consensus statement by the professional organizations of obstetricians/gynecologists, midwives, and nurses along with major donors.^1^

The International Confederation of Midwives (ICM) recently included PAC and postabortion family planning as basic skills in their “Essential Competencies for Basic Midwifery Practices”.^99^ Postabortion family planning should likewise be incorporated into emergency obstetrical care (EmOC) indicators for managing incomplete abortion.^100^

Health management information systems need to universally report postabortion contraceptive services as an essential measure of program and provider performance. Documentation should include the percentage of postabortion clients receiving contraceptives by method before leaving the facility and at return visits.

Documentation of method-specific uptake in the postabortion period needs a special focus when using misoprostol. Many of the misoprostol studies failed to report on this key component of PAC. LARCs deserve special emphasis, given that postabortion IUD insertion must be delayed until complete evacuation after misoprostol.

## POLICY, ADVOCACY, AND FUTURE RESEARCH

Among the many topics for future research, two especially stand out. Cost studies are needed to better assess the potential benefits of misoprostol in comparison with both vacuum aspiration and sharp curettage, especially given that sharp curettage is still widely used. Better evaluation is needed on the effects of misoprostol on expanding the scope of practice for nurses and midwives, frequency of surgical procedures, use of lower-level facilities, and community engagement.

For women wanting to use the IUD postabortion, there may be benefits to offering immediate postabortion insertion with vacuum aspiration as an alternative to delayed insertion with misoprostol and the lost-to-follow-up that inherently occurs—an opportunity for research.

Advocacy for universal access to postabortion family planning is supported by health professional organizations, major donors, and maternal health networks.^1^ In parallel, this alliance strongly advocates universal access to postpartum family planning.^38^ Together these advocacy and policy positions create a foundation for universal access to family planning in the postpartum and postabortion periods, with major implications for policy, practice, and training, both in-service and preservice.

## Notes

**Peer Reviewed**

**First Published Online:** 2016 Aug 25
